# Comparison of RNA extraction methods from biofilm samples of *Staphylococcus epidermidis*

**DOI:** 10.1186/1756-0500-4-572

**Published:** 2011-12-30

**Authors:** Angela França, Luís DR Melo, Nuno Cerca

**Affiliations:** 1 IBB-Institute for Biotechnology and Bioengineering, Centre of Biological Engineering, University of Minho, Campus de Gualtar, 4710-057 Braga, Portugal

## Abstract

**Background:**

Microbial biofilms are communities of bacteria adhered to a surface and surrounded by an extracellular polymeric matrix. Biofilms have been associated with increased antibiotic resistance and tolerance to the immune system. *Staphylococcus epidermidis *is the major bacterial species found in biofilm-related infections on indwelling medical devices. Obtaining high quality mRNA from biofilms is crucial to validate the transcriptional measurements associated with the switching to the biofilm mode of growth. Therefore, we selected three commercially available RNA extraction kits with distinct characteristics, including those using silica membrane or organic extraction methods, and enzymatic or mechanical cell lysis, and evaluated the RNA quality obtained from two distinct *S. epidermidis *bacterial biofilms.

**Results:**

RNA extracted using the different kits was evaluated for quantity, purity, integrity, and functionally. All kits were able to extract intact and functional total RNA from the biofilms generated from each *S. epidermidis *strain. The results demonstrated that the kit based on mechanical lysis and organic extraction (FastRNA^® ^Pro Blue) was the only one that was able to isolate pure and large quantities of RNA. Normalized expression of the *icaA *virulence gene showed that RNA extracted with PureLink™ had a significant lower concentration of *icaA *mRNA transcripts than the other kits tested.

**Conclusions:**

When working with complex samples, such as biofilms, that contain a high content extracellular polysaccharide and proteins, special care should be taken when selecting the appropriate RNA extraction system, in order to obtain accurate, reproducible, and biologically significant results. Among the RNA extraction kits tested, FastRNA^® ^Pro Blue was the best option for both *S. epidermidis *biofilms used.

## Background

*Staphylococcus epidermidis*, a normal inhabitant of normal human skin and mucosa, has recently emerged as a leading cause of biofilm-related infections, particularly, in patients with indwelling medical devices [1,2] due to its ability to adhere to abiotic surfaces and to form biofilms [2,3]. The quantification of specific messenger RNA (mRNA) from these biofilms is crucial to understanding the molecular mechanisms behind biofilm formation and maturation on the surface of medical devices. The success of any RNA-based analysis depends on the yield, purity, and integrity of the RNA [4,5]. However, different RNA extraction methods can yield RNA with varying levels of quality [6,7]. Currently, there are numerous methods for RNA extraction available, however there are only a few published studies comparing RNA extraction from biofilm samples [8-10]. Biofilm samples pose an increased problem to RNA extraction procedure mainly due to the presence of the extracellular matrix, which is estimated to comprise about 90% of the total biofilm biomass [11]. Polysaccharides, the major component of the *S. epidermidis *biofilm matrix, seems to interfere with RNA extraction methods making bacterial cell lysis and nucleic acid purification difficult, and the purified RNA may still contain inhibitory substances [12,13]. Therefore, in this study we compared three different commercially available RNA extraction kits to determine their ability to obtain high quantity, pure, intact, and functional RNA from *S. epidermidis *biofilms. The selected kits were based on distinct procedures and properties: FastRNA^® ^Pro Blue (MPBiomedicals, Irvine, CA, US) uses mechanical and chemical lysis together with organic extraction; PureZOL™ RNA isolation reagent (Bio-Rad, Hercules, CA, US) uses chemical lysis with organic extraction, while PureLink™ RNA Mini Kit (Invitrogen, San Diego, CA, US) uses enzymatic lysis and silica membrane extraction.

## Methods

### Bacteria and growth conditions

*S. epidermidis *biofilms from two *S. epidermidis *strains with different genetic backgrounds (1457 [14] and M187 [15]) were individually used to form separate biofilms. Strains were individually propagated by inoculating a single colony in 2 mL Tryptic Soy Broth (TSB) (Oxoid, Cambridge, UK) from plates not older than 2 days and grown at 37°C in a shaker rotator at 120 rpm for 24 (± 2) hours. Then, 10 μL of cell suspension was transferred to 2 mL of fresh TSB supplemented with 1% (w/v) of glucose to induce biofilm formation in a 24-well plate (Orange Scientific, Braine-L'Alleud, Belgium). The plate was incubated at 37°C with shaking at 100 rpm for 24 (± 2) hours. Prior to total RNA extraction, the culture media was removed and the biofilm was washed with 1 mL of NaCl 0.9% solution to remove planktonic cells. In order to count the total viable cells (CFUs/mL) within each *S. epidermidis *biofilm, the biofilms were resuspended in 1 mL of NaCl 0.9% solution and sonicated on ice for 10 s at 30 W. This procedure eliminates bacterial aggregates that do interfere with the CFUs counting [16]. Subsequently, 10-fold dilutions in 0.9% NaCl were performed and plated on Tryptic Soy Agar (Oxoid) plates. The plates were then incubated at 37°C overnight.

### Biofilm matrix composition

Biofilm total biomass, protein, and polysaccharide matrix content was determined as described previously [17]. Briefly, the biofilm suspension was sonicated for 30 s at 30 W and, subsequently, centrifuged at 10.500 *g *for 6 min at 4°C. This procedure did not reduced cell viability as determined by CFU counting [16]. The supernatants were then filtered through a 0.2 μm nitrocellulose filter and the proteins and polysaccharide content determined by Bicinchoninic Acid (BCA) protein assay [17] (G-Biosciences, MO, US) and Dubois method [18], respectively. The total biomass of the biofilms was determined by optical density at 595 nm. This experiment was performed in triplicates.

### RNA extraction and quality indicators

Total RNA was isolated according the manufacturer's instructions, with the following modification, when appropriate: cell lysis was performed using 15 mg/mL of lysozyme for 60 min at 37°C with. This optimization increased the yield of total RNA 2-4-fold (data not shown). The final total RNA fraction was obtained by eluting or suspending the RNA in 45 μL of DEPC-treated water. To digest contaminating DNA, DNase (Fermentas, Burlington, Ontario, Canada) treatment was performed by adding 5 μL (10×) of reaction buffer with MgCl_2 _and 2 μL DNase I to the extracted RNA and incubating the mixture at 37°C for 30 min. Subsequently, 5 μL of 25 mM EDTA was added and incubated at 65°C for 10 min to inactivate the DNase I. Each experiment was performed in triplicates. The concentration and purity of the total RNA was spectrometrically assessed using a NanoDrop 1000™ (Thermo Scientific, Waltham, MA, US). The absorbance ratios A_260_/A_280 _were used as indicators of protein contamination and A_260_/A_230 _as indicators of polysaccharide, phenol, and/or chaotropic salts contamination [19]. The integrity of the total RNA was assessed by visualization of the 23S/16S banding pattern. Electrophoresis was carried-out at 80 V for 60 min using a 1% agarose gel. The gel was stained with ethidium bromide and visualized using a GelDoc2000 (Bio-Rad). Total RNA extractions were performed two to four times.

### Real time PCR (qPCR)

To determine if the extracted RNA was functional, 40 ng of total RNA was reverse transcribed using an iScript™ cDNA Synthesis Kit (Bio-Rad) following the manufacturer's instructions. Real time PCR (qPCR) was performed to quantify the mRNA transcripts. Primers (Invitrogen), specific for 16S rRNA, reference gene, and *icaA*, a well-known virulence gene of *S. epidermidis *were designed using the Primer3 software [20] (Table 1). The experiment was performed using CFX96™ thermocycler (Bio-Rad) with the following cycling parameters: 30 s at 94°C followed by 40 repeats of 5 s at 94°C, 10 s at 60°C, and finally 15 s at 72°C, using Sso Fast™ Evagreen Supermix 2× mix (Bio-Rad). Primers efficiency was determined by the dilution method as well as performing a temperature gradient reaction from 50 to 65°C. At 60°C, both set of primers had the best and more similar efficiencies (98 ± 4% for *icaA *and 95 ± 7% for 16S *rRNA*). To ensure the absence of genomic DNA contamination, a negative control was included in the reverse transcriptase reaction. The cycle threshold detection of each gene was determined using the standards parameters of the software. The melting curves were evaluated to ensure the absence of unspecific products and primer dimer formation. The expression of *icaA *was determined by using the delta Ct method (2^ΔCt^), a variation of the Livak method, where ΔCt = Ct (reference gene)-Ct (target gene). The data analysis was based on two to four independent experiments.

**Table 1:** Primers used in cDNA synthesis and amplification by qPCR

Target gene		Primers sequence (5' to 3')
**16S**	FW	GGGCTACACACGTGCTACAA
	
	RV	GTACAAGACCCGGGAACGTA

***icaA***	FW	TGCACTCAATGAGGGAATCA
	
	RV	TAACTGCGCCTAATTTTGGATT

### Statistical analysis

Statistical significance of results was determined by unpaired *t *test using the Analysis Toolpak of Microsoft Excel 2007. *P *< 0.05 was considered to be statistically significant.

## Results and Discussion

**Figure 1:**
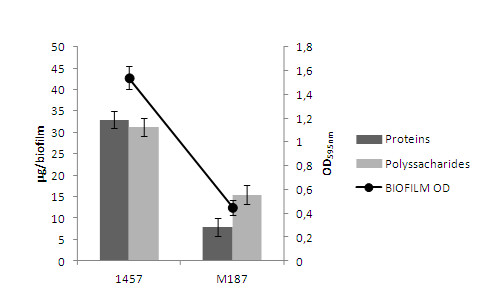
**Quantification of biofilm formation and matrix composition**. The biofilm forming capacity and protein and polysaccharide content in the biofilm matrix of each *S. epidermidis *strain. The optical density (OD 595 nm) indicates the biofilm-forming capacity of each *S. epidermidis *strain. The bars and the dot represent the mean plus or minus standard deviation of three independent experiments (*P < 0.01 between strains).

The success of any RNA-based analysis depends on the quantity, purity, and integrity of the RNA [4,5]. RNA quality is influenced by the sample's nature and by the principle of the RNA extraction kit used. Thus, our objective was to test the performance of three different commercially available RNA extraction kits when using *S. epidermidis *biofilms as a sample. Regarding the total RNA yield obtained by the different kits, FastRNA^® ^Pro Blue is clearly the kit with higher performance for both *S. epidermidis *strains (*P *< 0.01 unpaired *t*-test) (Table 2). Comparing the RNA purity, FastRNA^® ^Pro Blue was, again, the kit that extracted RNA with the highest purity. While most of the kits/strain combination produced total RNA with no protein contamination (A_260_/A_280_), the only kit with a polysaccharides level (A_260_/A_230_) above 1.8 was FastRNA^® ^Pro Blue (*P *< 0.01 unpaired *t*-test). Interestingly, significant differences were found in the total RNA quantity of the two strains of *S. epidermidis *used: while using FastRNA^® ^Pro Blue strain 1457 yielded more RNA than strain M187, the opposite occurred in all the remaining kits. These differences are probably related with the strain specific ability to form biofilm and also with the composition of the biofilm matrix (Figure 1). These strains were selected from a collection previously characterized for biofilm formation [21]: strain 1457 is a strong biofilm producer, while strain M187 is a moderate biofilm producer. When the biofilm matrices were extracted and analyzed, strain 1457 presented a significantly higher protein (*P *< 0.01 unpaired *t*-test) and polysaccharide (*P *< 0.05 unpaired *t*-test) content than strain M187. As strain 1457 had a thicker biofilm, it seems reasonable to assume that the total number of cells available per biofilm would be higher, even taking in consideration that the majority of biofilm is composed of matrix and not bacteria [11]. We have confirmed this by resuspending the biofilms in 0.9% NaCl, performed dilutions and plating in TSA. While biofilms from strain 1457 contained 4.2×10^9 ^CFU/mL, strain M187 had 6.3×10^8 ^CFU/mL. Thus, the initial amount of bacterial cells used for the RNA extraction seams to influence the total RNA quantity, as also described in the manufacturer guidelines. However, the relationship between the amount of sample and the RNA yield is not linear and each kit normally has an optimal range of sample concentration (PureLink™ - up to 1 × 10^9 ^bacteria, PureZOL™ - up to 2.4 × 10^9 ^bacteria, FastRNA^® ^Pro Blue- up to 1 × 10^9 ^bacteria). Nour et al. [6] extracted RNA from rabbit blood samples using different extraction kits and determined that in one of the extraction kits used, too much concentrated sample would result in lower yield. However, in the majority of the cases reported, a lower initial sample concentration, yields lower amounts of RNA [6]. In the specific case of biofilms, higher cell densities will also mean higher external protein and polysaccharide contents, as confirmed herein (Figure 1). The presence of the biofilm matrix did not interfere in the extraction process of FastRNA^® ^Pro Blue while in the remaining kits this was not the case. Interestingly, with strain M187, where the matrix presented lower levels of protein and polysaccharides, PureZOL™ and PureLink™ were more efficient as compared with strain 1457. Since FastRNA^® ^Pro Blue was the only kit using mechanical lysis and had the highest RNA extraction performance, we tried to perform RNA extraction with PureZOL™ and PureLink™ preceded with the mechanical lysis step present on FastRNA^® ^Pro Blue. Therefore, each biofilm was resuspended in the lysis buffer of the respective kit, but the lysis was completed mechanically following FastRNA^® ^Pro Blue instructions. Interestingly, this modification did not significantly increased the RNA yield (*P *> 0.10 unpaired *t*-test) (data not show). This suggests that the high RNA yield obtained with FastRNA^® ^Pro Blue depends not only on the mechanical lysis, but also on the chemistry. The buffer used for FastRNA^® ^Pro Blue lysis procedure contains phenol which is known to induce cell lysis [22], thus yielding more RNA than the other kits, which only used beads.

**Table 2:** Comparison of the RNA yield and purity obtained by the three RNA extraction kits

Extraction kit	Strain	RNA yield (ng/μl)	A_260_/A_280 _ratio	A_260_/A_230 _ratio
FastRNA^®^	1457	513 ± 135**	2.18 ± 0.06	2.06 ± 0.01**
	
	M187	359 ± 14**	2.09 ± 0.04	1.92 ± 0.06 **

PureZOL™	1457	18 ± 7	1.70 ± 0.06	0.30 ± 0.01
	
	M187	50 ± 7 *	1.70 ± 0.07	0.63 ± 0.16

PureLink™	1457	17 ± 3	1.99 ± 0.08	1.35 ± 0.04
	
	M187	30 ± 3*	2.10 ± 0.06	1.25 ± 0.63

The values represent the mean plus or minus standard deviation of 2 to 4 independent experiments (**P < 0.01 between kits; *P < 0.05 between strains)

Besides the yield and purity, the extracted RNA should be intact and functional, in order to proper analyze the quantification of gene expression [4]. RNA integrity was assessed by agarose gel electrophoresis as reported above, and the expected double banding pattern of the 23S/16S and the absence of a smear indicated the good integrity of RNA extracted (data not shown).

A previous study regarding the optimization of complementary DNA (cDNA) synthesis using commercially available kits, showed that in order to obtain reliable mRNA quantification at least 40 ng of total RNA is necessary [23]. Thus, for cDNA synthesis and subsequent qPCR analysis we have normalized the concentration of total RNA obtained by the different kits to 40 ng. The qPCR results presented in Table 3 clearly show differences between each RNA extraction kit. Since 16S rRNA is more abundant and stable, qPCR results will be highly influenced by the detection of specific mRNAs. This is of the upmost importance when using RNA for high throughput analysis, such as microarrays. Interestingly, the expression levels of *icaA *were significantly lower (*P *< 0.01 unpaired *t*-test) when using the RNA obtained from the PureLink™. This significant difference elucidates the importance of selecting the proper RNA extraction kit for the biological system being studied. It has been point-out that qPCR requires low amounts of total RNA, as when compared with microarrays [24]. However, the results reported here suggest that while qPCR can detect the expression of genes, independent of the quality and yield of RNA, the biological significance of the determined expression can be somewhat impaired. When trying to detect low expressing genes, a reduced RNA yield of extraction could place some low expressing genes below the limit of detection. While this was not the case with any of the kits tested in this study, however it can be inferred by the expression levels, that the kits with a lower total RNA yield were less efficient in recuperating mRNA.

**Table 3:** *icaA *expression values using RNA extracted from the three different kits

Extraction kit	Strain	16S rRNA Ct	*icaA *Ct	*icaA *expression
FastRNA^®^	1457	17.36 ± 0.41	31.48 ± 0.28	5.62 × 10^-5^
	
	M187	12.27 ± 0.81	27.07 ± 1.05	3.48 × 10^-5^

PureZOL™	1457	17.99 ± 0.30	31.84 ± 0.11	6.77 × 10^-5 ^
	
	M187	15.71 ± 0.83	29.58 ± 1.94	6.20 × 10^-5^

PureLink™	1457	15.25 ± 0.12	32.56 ± 0.11	0.62 × 10^-5 ^**
	
	M187	13.83 ± 0.67	31.13 ± 4.02	0.62 × 10^-5 ^**

Total RNA (40 ng) was used to synthesize cDNA for qPCR. The *icaA *expression was calculated by the delta Ct method (2^ΔCt^). The values represent the mean plus or minus standard deviation of 2 to 4 independent experiments (**P < 0.01).

## Conclusions

Observing the overall results it can be seen that depending on the RNA extraction kit used, the quantification of mRNA transcripts can be impaired. Interestingly, of all the parameters tested, RNA purity seemed to have a lower impact in inhibiting RNA quantification. The A_260/280 _and A_260/230 _ratios are just indicators of possible contaminants in the RNA sample. While some of the contaminants can interfere in the downstream applications, it seems that more than the concentration of contaminants, the nature of contaminants will impair RNA quantification. FastRNA^® ^Pro Blue showed the best results, while PureLink™ RNA mini kit was the worst kit for *S. epidermidis *biofilm samples.

When working with complex samples, such as biofilms, that contain a high content extracellular polysaccharide and proteins, special care should be taken when selecting the appropriate RNA extraction system, in order to obtain accurate, reproducible, and biologically significant results. Testing different systems and probing for well described gene expression conditions might elucidate some less apparent pitfalls of RNA extraction kits.

## List of Abbreviations

BCA: Bicinchoninic Acid; cDNA: complementary DNA; CFU: Colony-forming unit: Ct: cycle threshold; mRNA: messenger RNA; OD: optical density: qPCR: Real time PCR; TSB: Tryptic Soy Broth.

## Authors' contributions

AF carried-out all of the RNA related experiments and drafted the manuscript. LDRM performed the biofilm formation and characterization analysis and aided in drafting the manuscript. NC conceived the study, participated in its design and coordination, and aided in drafting the manuscript. All authors read and approved the final manuscript.

## Competing interests

The authors declare that they have no competing interests.
